# Biological Activity of Pulcherrimin from the *Meschnikowia pulcherrima* Clade

**DOI:** 10.3390/molecules27061855

**Published:** 2022-03-12

**Authors:** Dorota Kregiel, Maria Nowacka, Anna Rygala, Renáta Vadkertiová

**Affiliations:** 1 Department of Environmental Biotechnology, Faculty of Biotechnology and Food Sciences, Lodz University of Technology, Wolczanska 171/173, 90-530 Lodz, Poland; anna.rygala@p.lodz.pl; 2 Culture Collection of Yeasts, Institute of Chemistry, Slovak Academy of Sciences, Dúbravská Cesta 9, 845 38 Bratislava, Slovakia; renata.vadkertiova@savba.sk; 3 Centre of Molecular and Macromolecular Studies, Polish Academy of Sciences, Sienkiewicza 112, 90-363 Lodz, Poland; mnowacka@cbmm.lodz.pl

**Keywords:** *Metschnikowia*, pulcherrimin, water affinity, antibiofilm, protection, UV–C light, temperature

## Abstract

Pulcherrimin is a secondary metabolite of yeasts belonging to the *Metschnikowia pulcherrima* clade, and pulcherrimin formation is responsible for the antimicrobial action of its producers. Understanding the environmental function of this metabolite can provide insight into various microbial interactions and enables the efficient development of new effective bioproducts and methods. In this study, we evaluated the antimicrobial and antiadhesive action of yeast pulcherrimin, as well as its protective properties under selected stressful conditions. Classical microbiological plate methods, microscopy, and physico-chemical testing were used. The results show that pure pulcherrimin does not have antimicrobial properties, but its unique hydrophilic nature may hinder the adhesion of hydrophilic bacterial cells to abiotic surfaces. Pulcherrimin also proved to be a good cell protectant against UV–C radiation at both high and low temperatures.

## 1. Introduction

Yeasts are one of the most important groups of microorganisms for industry and life sciences research. The yeast *Saccharomyces cerevisiae* has been used to process food and alcoholic beverages for centuries and is the most extensively studied eukaryotic model organism [1]. Other yeasts, usually described as non-conventional, have become important only recently. Non-conventional yeasts include the species of the *Metschnikowia pulcherrima* clade [2]. Precise taxonomic characterization of the strains of this clade is difficult, due to the lack of knowledge regarding the morphological and physiological characteristics of the newly described species, and because of clear rDNA barcode gaps. Their taxonomic characterization is further hampered by incomplete biological (reproductive) isolation of the species: some strains may be hybridized, while others possess chimeric genome structures that may have evolved from interspecies hybrids (alloploid genome duplication) [3].

Various strains belonging to the *M. pulcherrima* clade produce a characteristic marrow-red pigment called pulcherrimin and exert antagonistic effects on many types of microorganisms. The production of insoluble pulcherrimin results in depletion of iron from the environment, making it unavailable for other microorganisms that need it for their growth [4]. 

Chemically, pulcherrimin is a ferric chelate or salt of pulcherriminic acid (2,5-diisobutyl-3,6-dihydroxypyrazine-1,4-dioxide), or a tautomeric form of that compound [5,6]. Pulcherrimin is very slightly soluble in water and insoluble in all organic solvents. However, it is easily soluble in strong alkaline solvents and remarkably resistant to relatively strong acids. The chemical analysis of pulcherrimin is very difficult, due to its chemical properties and structure. Recent methods for identifying pulcherrimin used NMR spectroscopy and Raman spectroscopy [6,7].

Pulcherrimin, a red iron chelate, is produced by some yeasts and bacteria. In natural or anthropized environments, pulcherrimin formation is part of the complex network of mechanisms and interactions that connect living organisms, including growth control or biofilm inhibition. It has been noted that the depletion of iron by pulcherriminic acid and the formation of insoluble pulcherrimin pigment are important action mechanisms of biocontrol [4,8,9,10]. Pulcherrimin-producing yeast species of genera: *Metschnikowia*, *Lipomyces*, *Kluyveromyces*, and *Dipodascopsis* are considered effective antimicrobial agents against various microorganisms, with great potential for biocontrol applications—particularly in the phyllosphere, where these yeasts are frequently found [3,4,6].

Over the last decade, there have been numerous studies on the biocontrol ability and action mechanisms of large numbers of strains belonging to the *M. pulcherrima* clade, isolated from various substrates [11,12,13]. It has also been observed that the amount of pulcherrimin formed varies considerably within strains and may be a function of environmental conditions [2,6,14,15]. The strong antagonistic properties of pulcherrimin-producing strains have encouraged research into the other possible roles of this metabolite in natural habitats. Is the role of yeast pulcherrimin limited to iron binding, or does it fulfil other environmental functions? The present study was aimed at finding other biological properties of yeast pulcherrimin.

## 2. Results

### 2.1. Antimicrobial Activity

**Figure 1:**
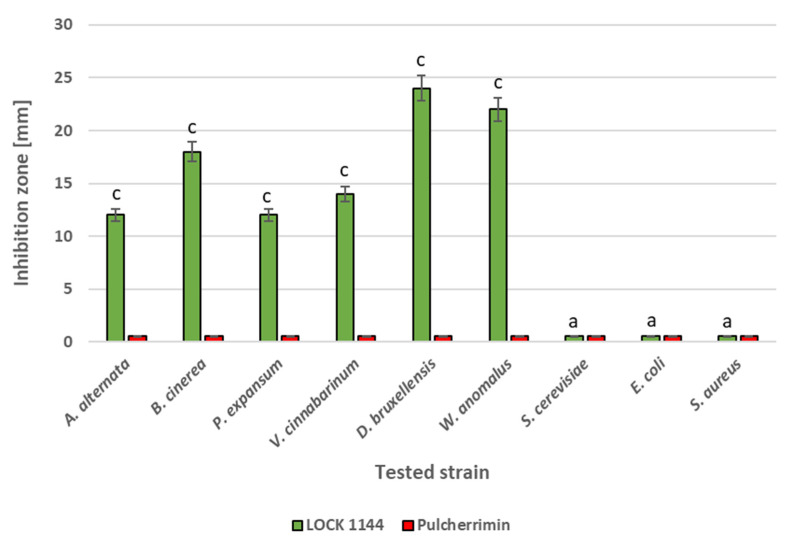
Comparison of antimicrobial activity of *Metschnikowia* sp. LOCK1144 cells and pure pulcherrimin suspension (~3% *w*/*v*) against tested microorganisms. Samples with pure pulcherrimine were taken as controls. Values show the mean ± standard deviation (SD, n = 3). Values with different letters are statistically different: ^a^: *p* ≥ 0.05; ^c^: *p* < 0.005.

The strain *Metschnikowia* sp. LOCK1144 showed antifungal activity against the molds *A. alternata*, *B. cinerea*, *P. expansum*, and *V. cinnabarinum*, which are common pathogens of fruit and vegetable crops. In addition, this strain showed antagonism against the non-conventional yeasts *D. bruxellensis* and *W. anomalus* (Figure 1).

However, it did not demonstrate antagonistic activity against either the classical yeast *S. cerevisiae* and the two bacterial strains *E. coli* (Gram-negative) and *S. aureus* (Gram-positive). Despite the antagonistic activity of the *Metschnikowia* LOCK1144 culture, pure pulcherrimin did not exhibit any antimicrobial activity.

### 2.2. Surface Free Energy of Pure Pulcherrimin

**Figure 2:**
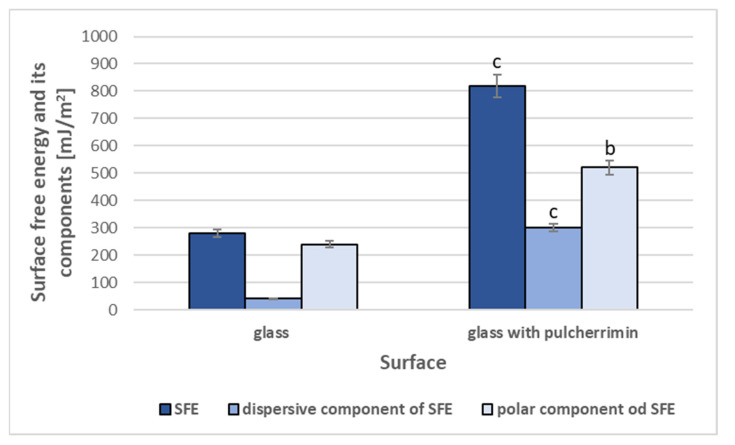
Surface free energy of glass and glass with yeast pulcherrimin evaluated for two solvents: a dispersive solvent (glycerol), and a polar solvent (water). Glass surface and its SFE components were taken as references. Values show the mean ± standard deviation (SD, n = 3). Values with different letters are statistically different: ^b^: 0.005 < *p* < 0.05; ^c^: *p* < 0.005.

The comparison of surface free energy SFE and its components for glass surface (control) and glass covered by pulcherrimin is given in Figure 2. The variant of glass with pulcherrimin yielded the highest ratios of both polar and dispersive components of the total surface free energy. Application of the pulcherrimin to the glass surface resulted in an almost 3-fold increase in the total SFE, which proves the remarkably strong hydrophilic properties of this yeast metabolite. 

Therefore, further experiments were conducted to evaluate the antiadhesive/antibiofilm activity of this compound.

### 2.3. Antibiofilm Activity

**Figure 3:**
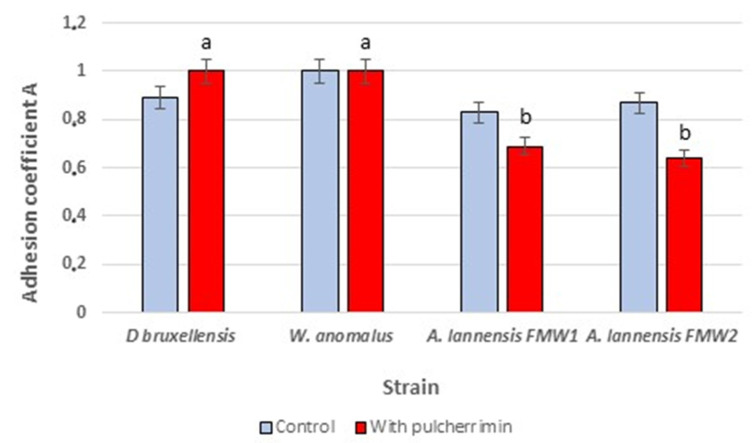
Adhesion coefficient A evaluated for microbial cultures incubated in culture media with and without pulcherrimin supplementation. Values show the mean ± standard deviation (SD, n = 3). Results obtained in minimal medium with pulcherrimin were compared with those for minimal medium without supplementation (control). Values with different letters are statistically different: ^a^: *p* ≥ 0.05; ^b^: 0.005 < *p* < 0.05.

**Figure 4:**
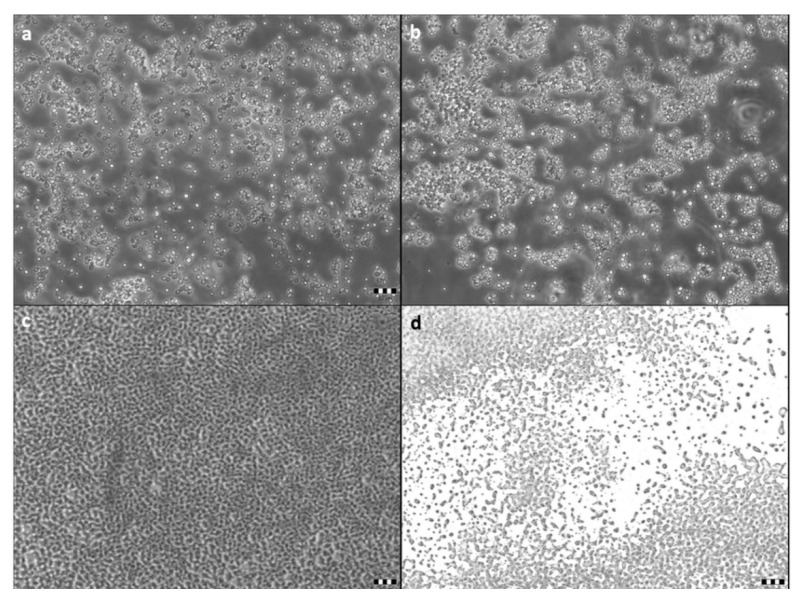
Attachment of microbial cells to glass surface. Yeast cells of W. anomalus cultivated without pulcherrimin (**a**) and with pulcherrimin (**b**). Bacterial cells of A. lannensis FMW2 cultivated without pulcherrimin (**c**) and with pulcherrimin (**d**). Bars represent 20 μm.

The antibiofilm properties of the pulcherrimin were investigated for two yeast strains, *D. bruxellensis* and *W. anomalus*, as well as two bacterial strains belonging to *Asaia lannensis*. These microorganisms were isolated from non-alcoholic beverages and showed strong adhesive properties [12,16]. The pulcherrimin in the culture medium did not affect the adhesion of the yeast cells, but slightly decreased attachment of the bacterial cells to the glass surface (Figure 3). These results were confirmed by microscopic studies (Figure 4).

### 2.4. UV-C Protection

Three *Metschnikowia* strains with different levels of pulcherrimin productivity were selected for this study [6]. In the absence of Fe(III) ions, the survival of the yeasts after UV treatment was very low and did not exceed 10%. However, cell survival was dependent on the pulcherrimin productivity of the yeasts. This parameter was the highest at 21% for the strain LOCK1144, which was the best producer of pulcherrimin (198 mg/L). The two other yeast strains produced smaller amounts of pulcherrimin and also exhibited lower cell survival (Table 1).

### 2.5. Heat Protection

**Figure 5:**
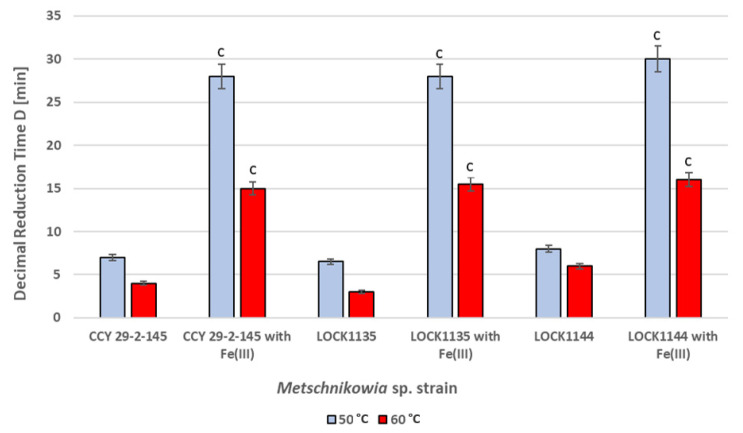
Decimal reduction times determined for three strains of *M. pulcherrima* clade: *M. pulcherrima* CCY 29-2-145, *Metschnikowia* sp. LOCK1135, and *Metschnikowia* sp. LOCK1144. Values show the mean ± standard deviation (SD, n = 3). Results obtained for minimal medium (control samples) were compared with those for minimal medium with Fe(III) ions (pulcherrimin production). Values with different letters are statistically different: ^c^: *p* < 0.005.

Figure 5 shows that the decimal reduction times (D) for all three cultures were significantly higher for the medium containing Fe(III) ions (pulcherrimin production) than the times obtained with the same culture medium but without iron supplementation. The decimal reduction times for the cells incubated at 50 °C (D_50_) ranged from 28 min to 30 min. They were about four times higher than those recorded for the control samples. The D_60_ values obtained for the minimal medium with Fe(III) at 60 °C were from 2.5 to 5 times higher than those with the medium without Fe(III). All these results prove the protective effect of pulcherrimin at higher temperatures.

### 2.6. Cryoprotection

**Figure 6:**
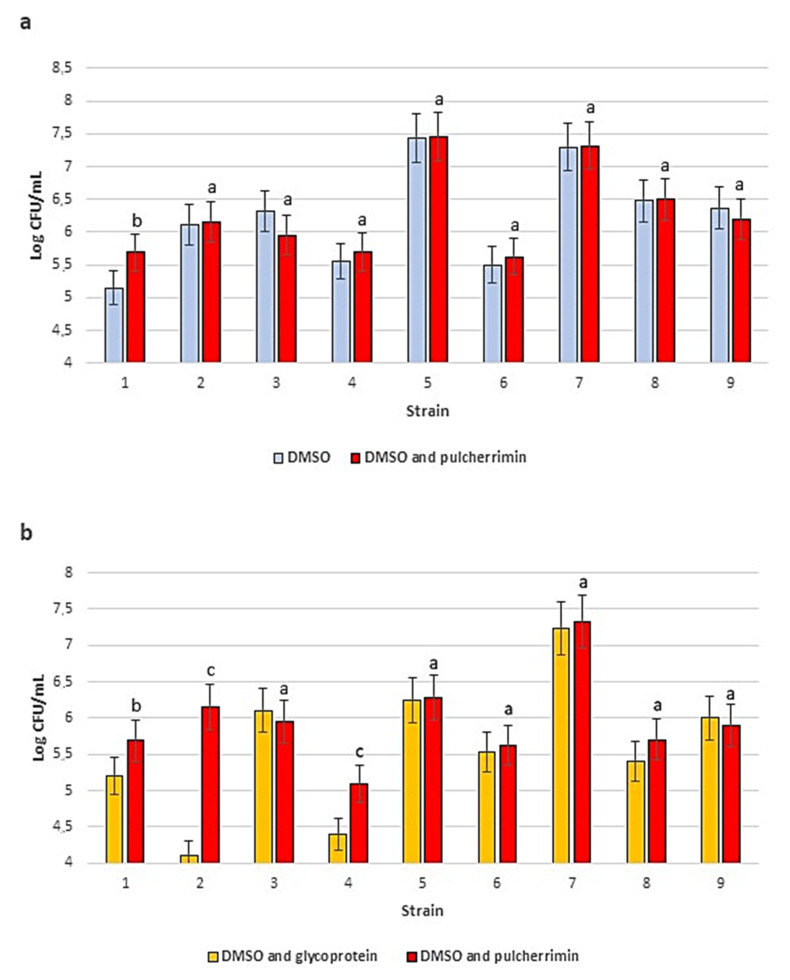
Comparison of the number of viable yeast cells after 6 months of storage at −196 °C in the presence of different cryoprotectants: DMSO (control) and DMSO with pulcherrimin (**a**); DMSO with glycoprotein (control) and DMSO with pulcherrimin (**b**). Yeast strains: 1—*C. macerans* CCY 10-1-17; 2—*G. candidum* CCY 16-1-25; 3—*P. flavescens* CCY 17-3-34; 4—*S. metaroseus* CCY 19-6-22; 5—*R. dairenensis* CCY 20-2-25; 6—*F. capsuligenum* CCY 29-143-1; 7—*C. zemplinina* CCY 20-178-1; 8—*A. porosum* CCY 30-18-5; 9—*S. pombe* CCY 44-1-3. Values with different letters are statistically different: ^a^: *p* ≥ 0.05; ^b^: 0.005 < *p* < 0.05; ^c^: *p* < 0.005.

The presence of pulcherrimin as the protective component in basal DMSO medium had a positive effect on the viability of most of the tested yeast strains. The results for this compound were comparable to the those obtained for the basal medium with DMSO and for the basal medium with DMSO and glycoprotein, which are routinely used in the CCY Collection (Figure 6).

**Figure 7:**
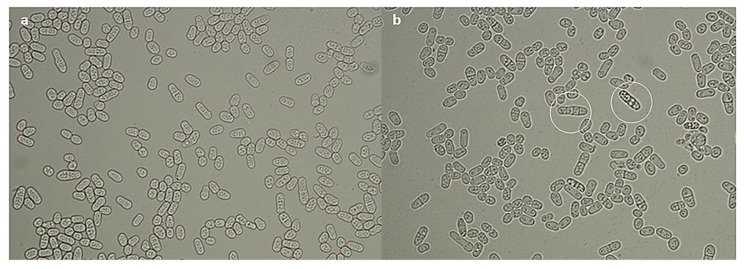
Morphology and division of *S. pombe* cells during cultivation in a culture medium: without pulcherrimin (**a**), with pulcherrimin (**b**). *S. pombe* cells show abnormal multiple divisions in the presence of pulcherrimin (white circles).

Nevertheless, for strains 1, 2 and 4 (*C. macerans*, *G. candidum*, and *S. metaroseus*), the presence of pulcherrimin had a clear positive impact on the survival of the yeast cultures after storage at −196 °C. Only in the case of strains 3 and 9 (*P. flavescens* and *S. pombe*) was a slight decrease in the number of viable cells observed, after 6 months of storage. Moreover, some cells of the strain *S. pombe* showed abnormal multiple cell divisions after thawing and incubation in the presence of pulcherrimin (Figure 7). The percentage of such abnormal cells did not exceed 5%, so it may be a coincidence. This phenomenon was no longer observed after subsequent passages in pulcherrimin-free culture media. Nevertheless, the phenomenon is worth additional research in the future.

## 3. Discussion

Yeast pulcherrimin, a red pigment produced by the *Metschnikowia pulcherrima* clade, is strongly connected with the biocontrol activity of yeast. However, the mechanisms by which it inhibits the growth of other microorganisms are still not well understood [2,4]. The iron-binding molecule pulcherrimin was described a century ago, but it is still not known which genes are responsible for its production [17]. The production of pulcherrimin is strongly dependent on the composition of culture media and other culture cultivation factors [6,14,18,19]. 

The recognition of pulcherrimin formation as an important biocontrol mechanism has stimulated research into its other possible ecological roles. According to the literature, pulcherrimin is an effective agent with various ecological roles. For example, pulcherrimin can control the growth of bacterial biofilms [20]. Pulcherrimin has also been reported to have antioxidant properties [21]. Due to its inhibition of urease activity, pulcherrimin can be used in agriculture as a component of fertilizers with controlled availability of urea [22]. Perhaps pulcherrimin, as an iron chelator, may affect photoprotection against cell damage caused by UVA radiation, as some Fe chelators display such property [23]. Encouraged by these findings, we began to investigate other potential roles of this metabolite in natural habitats. 

In our previous studies, we observed that pulcherrimin production is strain-dependent [6]. Therefore, in the present study, we used various strains of the *Meschnikowia pulcherrima* clade. Reference strains from culture collections and genetically characterized isolates were used that differed from each other in terms of pulcherrimin production [6]. In antimicrobial studies, we used the strain *Metschnikowia* sp. LOCK1144, which is the best producer of pulcherrimin. According to numerous studies in the literature, *Metschnikowia* sp. strains are especially active against molds and certain yeasts [4,11,12,13,24]. Despite the fact this strain showed inhibitory activity against the tested fungal strains, its purified red metabolite did not show any antimicrobial activity against either the fungal or bacterial strains. Therefore, we can confirm the hypothesis of Sipiczki and other researchers that the antimicrobial activity of *M. pulcherrima* strains depends mainly on the sequestration of iron by a pulcherrimin precursor (pulcherriminic acid), which leads to the depletion of this element from the environment [3,4,14,19].

There have been no previous studies on the surface properties of pulcherrimin. In the present study, we compared the surface energy of purified pulcherrimin to a reference hydrophilic glass surface. Hydrophilic materials have more thermodynamically favorable interactions with water and other polar solvents. They are commonly used in many applications, such as the separation of water from other materials, water binding, anti-fog coatings, and self-cleaning materials [25]. Pulcherrimin has a high affinity for water. This is a very interesting property from the standpoint of its ecological role. The remarkably hydrophilic properties of pulcherrimin prompted us to investigate the effect of the purified metabolite on the adhesion of microbial cells to glass. Research into glass/water systems is important for both the advancement of theoretical knowledge and practical applications. Although glass is widely believed to be an inert material, glass surfaces are slightly acidic and highly adsorptive, due to the presence of silanol groups (SiOH). These reactive groups interact via hydrogen bonding with other functional groups, allowing them to bond with the glass surface [26,27,28]. Glass as a carrier is less colonized than hydrophobic surfaces, so the colonization effect is usually obtained by strongly adhering microbial cells [29].

The antiadhesive properties of pure pulcherrimin were investigated against two yeast strains, *D. bruxellensis* and *W. anomalus*, and two bacterial strains belonging to *Asaia lannensis*. These microorganisms are members of the microbiota of non-alcoholic beverages, where they form consortia—cell aggregates with highly specific recognition and adhesion properties [16]. Our study found that hydrophilic pulcherrimin can reduce, but not prevent, cell adhesion. Bacterial *A. lannaensis* cells, previously described in the literature as hydrophilic, showed less adhesion to the glass surface in the presence of pulcherrimin [28]. No such effect was observed on the more hydrophobic yeast cells [16]. In the present study, pulcherrimin did not show a negative effect on yeast cell adhesion. 

In view of the hydrophilic properties of pulcherrimin, we decided to investigate its ability to function as a protective factor under stressful conditions. The maximum growth temperature of the *Metschnikowia* strains tested is 30 °C [12]. Pulcherrimin had a protective effect at temperatures above the maximum growth temperature. As a hydrophilic dipeptide that binds water, pulcherrimin can reduce the intensity of thermal denaturation processes, and thus acts as a thermal protectant. Yeast pulcherrimin may therefore act similarly to dehydrins—highly hydrophilic proteins that accumulate in plants during embryogenesis and under various environmental stresses [30].

Pulcherrimin can also act as a protectant in freezing processes. When biological materials freeze, so-called frozen-state degradation (predominantly aggregation) may occur. In many cases, this is linked to water crystallization inside and outside cells. Other factors, such as protein unfolding (due to either cold denaturation or the interaction of protein molecules with ice crystals) could also contribute to biological instability [31]. It is possible that the hydrophilic structure of pulcherrimin reduces the risk of mechanical damage by crystals forming during the freezing process. 

The addition of a small amount of pulcherrimin to the standard DMSO medium improved the survival of yeast strains after storage at the temperature of liquid nitrogen. Pulcherrimin protected the yeast cells like another biological cryoprotectant, yeast exoglycoprotein, which has been used in the CCY [32]. 

Pulcherrimin also shows other protective properties. It was found that the presence of this red pigment during UV irradiation significantly increased the viability of yeast cells. Bisset et al. and Karisma at al. have drawn attention previously to the role of iron chelates in the stabilization of homeostasis within cells during UV treatment [23,33]. Therefore, pulcherrimin may be considered an iron chelate for various protection strategies against the negative effects of UV radiation.

## 4. Materials and Methods

### 4.1. Microorganisms

The microorganisms used in the study were revitalized in our laboratories from stock cultures. Table 2 provides a list of the tested strains and their applications.

### 4.2. Pulcherrimin Production and Purification

Pulcherrimin was obtained from the *Metschnikowia* sp. LOCK 1144 culture in minimal broth [1% glucose (*w*/*v*), 0.3% (NH_4_)_2_SO_4_ (*w*/*v*), 0.1% KH_2_PO_4_ (*w*/*v*), 0.05% MgSO_4_ × 7H_2_O (*w*/*v*), 0.05% yeast extract (*w*/*v*), 0.05% FeCl_3_ (*w*/*v*)] after 48-h incubation on a rotary shaker (130 rpm) at 25 °C, according to a method described previously [6]. A 50-mL sample of each yeast culture was centrifuged at a temperature of 4 °C and speed of 5000× *g* for 10 min using a Centrifuge 5804R (Eppendorf, Hamburg, Germany). The precipitate containing the yeast cells and red pigment was treated with methanol (50 mL 99.8% methanol per 10 g of wet yeast biomass) at 4 °C. After treatment overnight, the yeast cells were centrifuged (4 °C, speed 5000× *g*, 10 min) and washed twice with distilled water (25 mL). The yeast biomass was re-suspended in 2M NaOH and centrifuged (4 °C, speed 5000× *g*, 10 min). The pH of the supernatant was adjusted to 1.0 using 4M HCl. The mixture was incubated at 100 °C for 30 min. The pigment precipitate was collected by centrifugation (4 °C, speed 8000× *g*, 20 min) and washed three times with 25 mL of distilled water. To obtain pure pulcherrimin, dissolution in NaOH and precipitation in HCl was repeated three times. Finally, the red pigment was collected by centrifugation and frozen at −20 °C. Quantitative determination of pulcherrimin was conducted spectrophotometrically at 410 nm.

### 4.3. Antimicrobial Activity

*E. coli* ATCC 8738 and *S. aureus* ATCC 6538 bacterial strains were grown in 10 mL TSB medium (Merck, Darmstadt, Germany) at 37 °C for 24 h. The cultures were diluted 100-fold, then 100 μL samples of the diluted bacterial cultures were spread on CASO agar plates (Merck, Darmstadt, Germany). Fungal suspensions were prepared in the same way. MEB broth was used for cultivation at 25 °C, then the obtained fungal cultures were spread on MEA plates (Merck, Darmstadt, Germany). When the surface of the microbial plates dried, 4 μL of pulcherrimin suspension (3% *w*/*v*) in water was dropped on each plate in triplicate. The plates with bacterial cultures were incubated at 37 °C for 48 h. The plates with fungal strains were incubated at 25 °C for 7 days. After incubation, the inhibition zones were measured manually and expressed in mm. The antagonistic effects of viable *Metschnikowia* sp. LOCK 1144 cells against the tested microbial species was examined, using the cross method with some modifications [12]. The obtained microbial suspensions were spread evenly on MEA agar (fungi) or CASO agar (bacteria) plates. Then, in duplicate, a *Metschnikowia* sp. cell suspension (~10^7^ cfu/mL, 4 μL) was dropped on the plates, which were incubated at 25 °C for 2–7 days (fungi) or 37 °C for 48 h (bacteria). Inhibition zones were measured (mm) at the end of the incubation period. Plates inoculated by the *Metschnikowia* LOCK 1144 strain were used as control samples for the series treated by the yeast pulcherrimin.

### 4.4. Surface Free Energy Measurements

Pulcherrimin was spread as a thin surface film on commercially available clean glass slides (Lab Glass, Löberöd, Sweden) using a slit coating applicator (film thickness 150 µm). The surface free energy of the obtained material was estimated by contact-angle measurements (sessile drop technique) at the film–air interface, using deionized water and glycerol (Chempur, pure p.a., anhydrous) as reference liquids. Surface energy, including polar and dispersive components, were estimated by the Owens–Wendt method [27,34]. Glass slides without pulcherrimin were used as control samples.

### 4.5. Antibiofilm Activity

The experiments were performed with *D. bruxellensis* NCYC D5300, *W. anomalus* NCYC D5299, and *A. lannensis* FMW1 microbial strains isolated from soft drinks in Poland [16,35]. The strains were cultivated in a minimal medium [2% saccharose (*w*/*v*), 0.1% (NH_4_)_2_SO_4_ (*w*/*v*), 0.3% KH_2_PO_4_ (*w*/*v*), 0.2% MgSO_4_ × 7H_2_O (*w*/*v*), 0.05% yeast extract (*w*/*v*)] with 0.25% (*w*/*v*) pulcherrimin. The medium without pulcherrimin was used as control. The culture medium (20 mL) was poured into 25 mL Erlenmeyer flasks with the sterile glass carriers (Star Frost 76 × 26 mm, Knittel Glass, Germany). The amount of inoculum was standardized to obtain a microbial cell concentration in the culture medium of 100–1000 CFU/mL at the beginning of the experiment. The microbial cultures were incubated at 25 °C on a laboratory shaker (115 rpm) for 7 days. Cell adhesion to the carriers was analyzed luminometrically and by microscopic observations. For luminometric tests, the carrier plate was removed from the culture medium, rinsed with sterile distilled water and swabbed using free-ATP sampling pens (Merck, Darmstadt, Germany). Each measurement was reported in relative light units (RLU), using an HY-LiTE2 luminometer (Merck, Darmstadt, Germany). Additionally, the relative adhesion coefficient (A) was calculated according to the following formula:

Adhesion coefficient A = number of adhered cells [RLU]/number of planktonic cells [RLU](1)

In microscopic studies, the adhered microbial cells were stained with basic fuchsin (0.5% *w*/*v*). The cells on the carrier were observed using an OLYMPUS BX41 light microscope with a DP72 digital camera [27].

### 4.6. UV-C Protection

Three strains of the *M. pulcherrima* clade were used in the experiments: *M. pulcherrima* CCY 29-2-145, *Metschnikowia* sp. LOCK 1135, and *Metschnikowia* sp. LOCK 1144. These strains were selected on the basis of differences in the amounts of pulcherrimin they produce. Cultivation in the minimal medium with Fe(III) ions was continued at 25 °C for 72 h [6]. Yeast cultures were also obtained under the same conditions, but in media without FeCl_3_ supplementation. These cultures were used as control samples. 

Before UV–C treatment, yeast cell cultures (30 mL, ~1 × 10^8^ CFU/mL) were transferred to 90-mm sterile plastic Petri dishes. A 1.25-cm magnetic stir bar was placed at the center of each dish to introduce turbulent flow during UV–C exposure. An average liquid sample thickness of 5 mm was recorded. Before UV–C tests, any negative influence of the magnetic stirrer on yeast cell viability was checked and ruled out. The cultures were then subjected to UV–C irradiation by placing each plate under a 15-W mercury lamp (Sankyo, Denki, Japan) [36]. The distance of the lamp to the sample surface was 25 cm. During UV–C exposure, the yeast cultures were stirred (1000 rpm) to keep turbulent flow for 5 min. After radiation, the samples were left for photoreactivation at room temperature for 60 min in the dark. The yeast samples were subjected to quantitative microbiological tests both before UV–C treatment and after radiation and photoreactivation, using the classical plate count method on YPD agar (Merck, Darmstadt, Germany). The inoculated plates were incubated for 72 h at 25 °C. The results were expressed as colony forming units per mL (CFU/mL), taking into account the appropriate dilutions. The survival of the yeast cells was calculated according to the following formula:

Survival [%] = [1 − (ΔN/N_o_)] × 100% (2)

where ΔN is the difference in the number of viable cells before and after treatment [CFU/mL], and N_o_ is the initial number of cells [CFU/mL].

### 4.7. Heat Protection

The three strains of the *M. pulcherrima* clade used in this experiment (CCY 29-2-145, LOCK 1135, and LOCK 1144) differed in terms of the amounts of pulcherrimin they produced. The cultures were cultivated in a minimal medium with Fe(III) ions at 25 °C for 72 h. Yeast cultures grown under the same cultivation conditions, but without supplementation with FeCl_3_, were used as control samples. Prior to heat treatment, the yeast suspensions (3 mL, ~1 × 10^8^ CFU/mL) were transferred to sterile glass tubes, which were placed in water baths (50 °C, 60 °C). Temperature measurements inside the control glass tubes were performed using a mercury thermometer. After heat treatment (time from 5 min to 60 min), consecutive samples from each culture were immediately transferred to an ice bath for cooling. The final stage of the study was the quantitative determination of viable yeast cells using the plate count method and YPD agar. The results were expressed as colony forming units per ml [CFU/mL], taking into account the appropriate dilutions. The decimal reduction times (D_50_, D_60_) were determined mathematically for each yeast strain and temperature from the equation:

D [min] = t/(log N_o_ − log N_t_) (3)

where N_o_ is the initial number of living cells [CFU/mL], and N_t_ is the surviving population [CFU/mL] after an exposure time t [min] [37].

### 4.8. Cryoprotection

Research on the protective effect of the yeast pulcherrimin was carried out at the Culture Collection of Yeasts (CCY), Institute of Chemistry, Slovak Academy of Sciences, Bratislava. Nine different yeast strains were tested for their survival rates after storage in liquid nitrogen at −196 °C for 6 months. The set of strains included: *Cystofilobasidium macerans* CCY 10-1-17, *Sporidiobolus metaroseus* CCY 19-6-22, and *Rhodotorula dairenensis* CCY 20-2-25, which produce carotenoid pigments; *Papiliotrema flavescens* CCY 17-3-34, which produces extracellular polysaccharides; two yeast-like species, *Galactomyces candidum* CCY 16-1-25 and *Apiotrichum porosum* CCY 30-18-5; two osmotolerant yeasts, *Filobasidium capsuligenum* CCY 29-143-1 and *Candida zemplinina* CCY 29-178-1; *Schizosaccharomyces pombe* CCY 44-1-3, which reproduces by fission [38,39,40] (Table 2).

A basal cryomedium consisting of 7% wort extract (*w*/*v*), 0.3% yeast extract Difco (*w*/*v*), and 0.5% peptone Difco (*w*/*v*) was sterilized by autoclaving at 121 °C for 20 min. Three series of samples were prepared. The first consisted of 0.5 mL of the basal medium, 0.1 mL of an individual cell suspension (10^7^–10^8^ CFU/mL), and the penetrating cryoptotective agent DMSO (Fluka, Buchs, Switzerland), added to a final concentration of 10% (*v*/*v*) (control). The second series was prepared in the same way as the first series but was supplemented with a non-penetrating cryoprotective agent—the extracellular yeast glycoprotein, which was added to a final concentration of 0.27% (*w*/*v*). Glycoprotein was obtained using the method of development in CCY [32]. The third series was again prepared in the same way but was supplemented with the yeast pulcherrimin to a final concentration of 0.006% (*w*/*v*). The pulcherrimine concentration was chosen after series of preliminary experiments in the CCY Collection (unpublished data). Cryogenic screw-cap vials (Merck, Darmstadt, Germany) were used to store the yeast cultures in liquid nitrogen.

After 6 months of storage in liquid nitrogen, the vials were thawed in a water bath (37 °C) for 30 min. The yeast cultures were recultivated on either malt extract agar (Merck, Darmstadt, Germany) or YPD agar (Merck, Darmstadt, Germany). Quantitative evaluation of yeast growth was performed using the plate count method after incubation for 48–72 h at optimal temperatures for the growth of the individual yeast cultures. The numbers of yeast cells that survived were calculated and expressed as CFU/mL. The first series (DMSO) and the second series (DMSO and yeast glycoprotein) were used as the controls for the third series (DMSO and the yeast pulcherrimin). Following storage of the yeast cells mixed with pulcherrimin in liquid nitrogen, the morphology of the survived yeast cells was examined microscopically (Nikon Eclipse 80i).

### 4.9. Statistics

The results were presented as the mean ± SD of three separate experiments. The mean value results were compared using one-way repeated measures analysis of variance (ANOVA; OriginPro 8.1, OriginLab Corp., Northampton, MA, USA). Values with different letters are statistically different: ^a^: *p* ≥ 0.05; ^b^: 0.005 < *p* < 0.05; ^c^: *p* < 0.005.

## 5. Conclusions

The present study confirmed further data on the antagonistic activity of the *Metschnikowia pulcherrima* clade. Although pulcherrimin itself does not exhibit antimicrobial activity, production of this metabolite leads to depletion of iron from the environment, which is the major mechanism of antimicrobial activity of the pulcherrimin producers. We demonstrated that the unique hydrophilic nature of pulcherrimin may hinder the adhesion of hydrophilic bacterial cells to abiotic surfaces. This yeast metabolite also proved to be a good cell protectant against UV–C radiation and temperature stress. This study provided new insights into the multifunctionality of pulcherrimin that could contribute to new applications of this yeast metabolite.

## Figures and Tables

**Table 1:** Survival of yeast cells after exposure to UV-C radiation.

*Metschnikowia* Strain	Fe(III) Ion Supplementation	Cell Number [CFU/mL]		Survival [%]
		Initial	After UV-C Treatment	
*Metschnikowia* sp. LOCK1135 ^1^	without	1.7 ± 0.2 × 10^8^	4.0 ± 0.3 × 10^6^	2.4 ± 0.2
	with (pulcherrimin formation)	1.0 ± 0.1 × 10^8^	1.3 ± 0.1 × 10^7^	12.0 ± 0.1 ^c^
*M. pulcherrima* CCY 29-2-145 ^2^	without	1.7 ± 0.2 × 10^7^	1.6 ± 0.4 × 10^6^	9.7 ± 3.2
	with (pulcherrimin formation)	3.4 ± 0.1 × 10^7^	5.2 ±0.3 × 10^6^	15.3 ± 0.5 ^b^
*Metschnikowia* sp. LOCK1144 ^3^	without	3.0 ± 0.3 × 10^8^	2.6 ± 0.1 × 10^7^	8.7 ± 0.5
	with (pulcherrimin formation)	3.3 ± 0.4 × 10^8^	7.0 ± 0.4 × 10^7^	21.0 ± 1.4 ^c^

^1^ weak pulcherrimin productivity 46 mg/L; ^2^ moderate pulcherrimin productivity 148 mg/L; ^3^ high pulcherrimin productivity 198 mg/L [6]. Results obtained in minimal medium with Fe(III) ions were compared with those for minimal medium without Fe(III) supplementation (control). Values with different letters are statistically different: ^b^: 0.005 < *p* < 0.05; ^c^: *p* < 0.005.

**Table 2:** Microbial strains used in the studies.

Microbial Strain	Applicable to the Testing
*Metschnikowia* sp. LOCK * 1144	Pulcherrimin production
*Metschnikowia* sp. LOCK 1144	Antimicrobial activity
*Escherichia coli* ATCC ** 8738	
*Staphylococcus aureus* ATCC 6538	
*Saccharomyces cerevisiae* LOCK 203	
*Dekkera bruxellensis* NCYC *** D5300	
*Wickerhamomyces anomalus* NCYC D5299	
*Alternaria alternata* LOCK 409	
*Botritis cinerea* LOCK 453	
*Penicillium expansum* LOCK 535	
*Verticillium cinnabarinum* LOCK 576	
*Metschnikowia* sp. LOCK 1144	Antibiofilm activity
*Dekkera bruxellensis* NCYC D5300	
*Wickerhamomyces anomalus* NCYC D5299	
*Asaia lannensis* FMW1	
*Asaia lannensis* FMW2	
*Metschnikowia pulcherrima* CCY **** 29-2-145	UV–C protection Heat protection
*Metschnikowia* sp. LOCK 1135	
*Metschnikowia* sp. LOCK 1144	
*Metschnikowia* sp. LOCK 1144	Cryoprotection
*Cystofilobasidium macerans* CCY 10-1-17	
*Galactomyces candidum* CCY 16-1-25	
*Papiliotrema flavescens* CCY 17-3-34	
*Sporidiobolus metaroseus* CCY 19-6-22	
*Rhodotorula dairenensis* CCY 20-2-25	
*Filobasidium capsuligenum* CCY 29-143-1	
*Candida zemplinina* CCY 29-178-1	
*Apiotrichum porosum* CCY 30-18-5	
*Schizosaccharomyces pombe* CCY 44-1-3	

The names of pulcherrimin producers are bolded. Abbreviation of collection name: (*) Lodz Culture Collection, Poland (LOCK); (**) American Type Culture Collection (ATCC); (***) National Collection of Yeast Cultures, UK (NCYC); (****) Culture Collection of Yeasts, Slovakia (CCY).

## Sample Availability

Samples of the compounds are not available from the authors upon request.
